# Modulation of Extracellular ATP Content of Mast Cells and DRG Neurons by Irradiation: Studies on Underlying Mechanism of Low-Level-Laser Therapy

**DOI:** 10.1155/2015/630361

**Published:** 2015-01-27

**Authors:** Lina Wang, Lei Hu, Ryszard Grygorczyk, Xueyong Shen, Wolfgang Schwarz

**Affiliations:** ^1^Acupuncture and Moxibustion College, Shanghai University of Traditional Chinese Medicine, 1200 Cailun Road, Shanghai 201203, China; ^2^Shanghai Research Center for Acupuncture and Meridians, 199 Guoshoujing Road, Shanghai 201203, China; ^3^Research Centre, Centre Hospitalier de l'Université de Montréal (CRCHUM), Tour Viger, 900 rue Saint-Denis, Montréal, QC, Canada H2X 0A9; ^4^Department of Medicine, Université de Montréal, CP 6128 Succursale Centre-Ville, Montréal, QC, Canada H3C 3T5; ^5^Institute for Biophysics, Goethe-University Frankfurt, Max von Laue Straße 1, 60438 Frankfurt am Main, Germany

## Abstract

Low-level-laser therapy (LLLT) is an effective complementary treatment, especially for anti-inflammation and wound healing in which dermis or mucus mast cells (MCs) are involved. In periphery, MCs crosstalk with neurons via purinergic signals and participate in various physiological and pathophysiological processes. Whether extracellular ATP, an important purine in purinergic signaling, of MCs and neurons could be modulated by irradiation remains unknown. In this study, effects of red-laser irradiation on extracellular ATP content of MCs and dorsal root ganglia (DRG) neurons were investigated and underlying mechanisms were explored *in vitro*. Our results show that irradiation led to elevation of extracellular ATP level in the human mast cell line HMC-1 in a dose-dependent manner, which was accompanied by elevation of intracellular ATP content, an indicator for ATP synthesis, together with [Ca^2+^]_i_ elevation, a trigger signal for exocytotic ATP release. In contrast to MCs, irradiation attenuated the extracellular ATP content of neurons, which could be abolished by ARL 67156, a nonspecific ecto-ATPases inhibitor. Our results suggest that irradiation potentiates extracellular ATP of MCs by promoting ATP synthesis and release and attenuates extracellular ATP of neurons by upregulating ecto-ATPase activity. The opposite responses of these two cell types indicate complex mechanisms underlying LLLT.

## 1. Introduction

Low-level-laser therapy (LLLT) is an increasingly used and effective complementary treatment in clinic, especially for wound healing, anti-inflammation, and pain relief. Mast cells (MCs), the vital immune cells, in dermis paly necessary roles in the process of wound healing [1]. MCs have recently been identified to have important anti-inflammatory functions* in vivo* to some extent [2], despite their well-known role as promoter of inflammation. Subcutaneous or mucosal MCs* in vivo* have been suggested to be involved in anti-inflammatory [3] and wound healing [4–7] reactions of LLLT. Recently, subcutaneous MCs have been shown to participate in the initial process of pain relief by laser acupuncture [8], an increasingly and frequently applied version of LLLT. In medicine, red and near-infrared (NIR) lasers, with wavelengths between 600 and 1,000 nm, are frequently applied in LLLT because laser light at these wavelengths can penetrate tissues in the millimeter range as absorption by human skin is low [9]. The approximate skin transmission depth of red light is about 1.5–2 mm [10], mainly reaching MCs residing in cutaneous and subcutaneous skin layers [11]. MCs in skin may, therefore, participate in LLLT mechanisms by being directly activated during red-laser irradiation. This hypothesis has been supported by some investigations* in vitro*: activation of degranulation [12, 13], [Ca^2+^]_i_ elevation [12–14], as well as triggering of whole-cell membrane currents [12] by red-laser irradiation were demonstrated in MCs.

Accumulating evidences suggest that purinergic signaling participates in various physiological and pathophysiological processes. Extracellular nucleotides are important players in regulating inflammatory responses through binding to purinergic P2 receptors present on all inflammatory cells, including MCs [15]. Activation of P1 receptors by adenosine or other agonists can promote the wound healing process [16]. Extracellular ATP is not only the endogenous agonist for several P2 receptors but also the precursor of other related nucleotides. The effect of irradiation on extracellular ATP content of MCs is unknown. In the periphery, MCs morphologically [17] and functionally [18] interact with nerve endings, in which purinergic signaling has a major role [19], and a crosstalk between MCs and peripheral neurons via purinergic signals exists [20]. Whether this crosstalk could be modulated by low-level irradiation remains unknown too.

In the present study, we assessed the responses of extracellular ATP content of MCs and DRG neurons to low-level red-laser irradiation and further explored the underlying mechanisms. The aim of this study is to better understand of the role of ATP purinergic signaling in LLLT effects at the cellular level.

## 2. Materials and Methods

### 2.1. Cells Cultivation and Isolation

The human mast cell line HMC-1 was kindly provided by Dr. J. H. Butterfield (Mayo Clinic, Rochester, MN, USA) and cultured as described previously [21]. In brief, the cells were incubated in phenol-red-free IMDM medium, supplemented with 2 mM L-glutamine, 25 mM HEPES, 10% (v/v) fetal bovine serum 1% penicillin, and streptomycin, in a 95% humidity-controlled incubator (Model: 310, Thermo, Thermo electron, Waltham, USA) with 5% CO_2_ at 37°C. Cell density was about 3 × 10^5^/mL.

Isolation of rat peritoneal MCs was modified according to Jensen et al. (2006) [22]. Briefly, adult Sprague-Dawley rats (280–320 g) were sacrificed by CO_2_ asphyxiation. 30 mL Ca/Mg free hank's balanced salt solution (HBSS) was injected into peritoneal cavity of each rat. After vigorous agitation of the abdominal area for 2 min, injected HBSS was collected and centrifuged at 400 g for 5 min with swing-out rotors. Ammonium-chloride-potassium lysing buffer (150 mM NH_4_Cl, 10 mM KHCO_3_, and 0.1 mM EDTA-Na_2_) was used to break erythrocytes. The cell pellet was well resuspended with 8 mL 70% isotonic percoll and gently overlaid with 2 mL RPMI 1640 medium. MCs stayed at the bottom fraction after centrifugation of the cell suspension at 580 g for 15 min. The MC pellet was resuspended in RPMI 1640 medium. The viability and the purity were identified with 0.4% trypan blue and 0.5% toluidine blue (TB). The cell density was adjusted as needed (see Section 2.6) and cells were equilibrated in the incubator for 2 h.

Isolation of rat DRG neurons was modified according to Burkey et al. (2004) [23]. Briefly, adult Sprague-Dawley rats (150–170 g) were sacrificed by CO_2_ asphyxiation. Hibateral DRGs were dissected from thoracic to lumbar regions of the vertebral column and placed in oxygenated Ca/Mg free HBSS. The connective tissue sheath around the ganglia was removed and then incubated in 3 mL of F12 medium containing 1.25 mg/mL collagenase IA, 300 U deoxyribonuclease IV (DNase), and 0.05% trypsin IX-S at 37°C for 50 min. At the end of the incubation period, 8 mL F12 medium containing 10% horse serum was added to terminate the enzymatic reaction. Ganglia pellet was collected by centrifugation (200 g, 2 min), resuspended in F12 medium, and mechanically agitated through a fire-polished glass Pasteur pipet until the suspension of dissociated cells was homogeneous. Nerve fragments were discarded by centrifugation at 60 g for 2 min, and isolated individual cells of the bottom pellet were resuspended in F12 medium once more and transferred to a 35 mm petri dish to culture for 2 h. At the end of the incubation, after nonneuronal cells were attached to the bottom, the unattached sensory neurons were collected for equilibration in the incubator for 2 h.

### 2.2. Skin Tissue Preparation

4-week-old male Sprague-Dawley rats (Shanghai Experimental Animal Center of Chinese Academy of Sciences, Shanghai, China) weighing 120–150 g were deeply anesthetized with ether. Each animal was placed in an airtight container with several cotton balls soaked with ether (Sinopharm Chemical Reagent Co., Ltd., Shanghai, China). The rats were not decapitated until they were deeply anesthetized. Hair in the area of the lateral tibia was shaved, and the skin was cut open with scissors to expose connective tissues, which were acutely separated with blunt forceps and scissors before incubation in bath solution (BS) (see Section 2.3) for 1 h. Tissue slices comprised three connective layers, with one loose layer sandwiched between two dense layers. The loose connective tissue was exposed by stretching the two dense layers in opposite direction. Thereafter, the tissue slices were fixed by tiny needles to hot glue support on the chamber bottom. To identify MCs, fixed slices were incubated in 0.5% TB for 10 min and subsequently washed 3 times with 95% ethanol. The present study was performed in accordance with the guidelines of the Animal Care and Use Committee of Shanghai University of Traditional Chinese Medicine (Certificate No. 2013012).

### 2.3. Reagents and Solutions

The BS for tissue slices contained (in mM) 137 NaCl, 2.7 KCl, 2 CaCl_2_, 5 MgCl_2_, 5.6 glucose, and 10 HEPES, pH 7.4 (adjusted with NaOH). The BS for HMC-1 cells was comprised of the following (in mM): 150 NaCl, 5 KCl, 2 CaCl_2_, 5 MgCl_2_, 4D-sorbitol, and 10 HEPES, pH 7.4 (adjusted with NaOH). Osmolality of the solutions was 310 mOsm/kg, as verified with osmometer (Model: 3300, Micro Osmometer, Advanced Instruments Inc., Norwood, MA, USA). All experiments were performed at room temperature (23–26°C).

Culture mediums, HBSS, serum, antibiotic, calcium green-1 AM, and pluronic F-127 were bought from Invitrogen Company (USA); all other chemicals were purchased from Sigma Company (USA).

Reagents were prepared in stock solutions and kept at −20°C and diluted in BS or culture medium before use. In some ATP-accessing experiments, HMC-1 cells were pretreated with NEM or BAPTA or ARL 67156 for 20 min before luminescence measurements.

### 2.4. Irradiation

According to the Kubelka-Munk theory [24], red-light irradiance decreases by >90% at a depth of 1.5–2 mm (see also [10, 25]). Hence, in our present experiments, we started with a dose of 17 J/cm^2^, 5% of the dose level used in our previous animal studies [8]. A higher light dose was achieved by prolonging exposure time. The laser device and irradiation parameters used for HMC-1 cells and tissue slice are presented in Table 1. For rat peritoneal MCs and neurons, the laser power density was adjusted to 18 mW/cm^2^, and then 20 min exposure generated 21 J/cm^2^.

In ATP-related experiments, equilibrated cell suspensions in 1.5 mL Eppendorf tubes (see Section 2.6) were exposed to laser light entering the tube vertically, in order to expose the entire suspension to irradiation. In light and fluorescence imaging experiments, light was introduced at 60-degree angle to tissue slices or HMC-1 cells, and all cells viewed under microscope were irradiated.

To monitor possible heating effects of laser irradiation, temperature was recorded by thermocouple. Temperature changes of perfusion BS induced by red-laser amounted to only 0.2°C during 15 min irradiation.

In order to exclude the possibility that reagents were modified by the laser light, the absorption spectrums of 100 *μ*M NEM, 25 *μ*M BAPTA, and 100 *μ*M ARL 67156 were scanned. None of the drugs showed significant absorbance at the 657 nm laser light.

### 2.5. Light and Fluorescence Images

To investigate the effects of laser irradiation on rat skin MC degranulation, tissue slices were fixed in a perfusion chamber and micrographs were captured under upright light microscope (Model: NF1, Nikon, Japan).

To estimate the [Ca^2+^]_i_ of HMC-1 cells, calcium green-1 fluorescence was measured as described previously [21]. Briefly, HMC-1 cells were grown on glass cover-slips coated with poly-L-lysine before mounting in the perfusion chamber. 5 mM calcium green-1 AM stock solution was dissolved in 20% (w/v) pluronic F-127. HMC-1 cells were loaded in phenol-red-free IMDM containing 4 *μ*M calcium green-1 AM for 45 min; then the loaded cells were superfused with BS. All solutions used in the fluorescence experiments contained 2.5 mM probenecid. Micrographs were captured under inverted light microscope (Model: TE2000-U, Nikon, Japan) by CCD video camera (Orca-ER, Hamamatsu photonics, Hamamatsu, Shizuoka, Japan). Images were digitized and averaged (3 frames), background corrected, and analyzed by an image-processing system (Wasabi software, Hamamatsu photonics, Hamamatsu, Shizuoka, Japan). Fluorescence intensities of individual cells in the viewing field were ascertained by averaging the image intensities collected from regions of interest within each cell. Graphs were colored by Image J software (National Institutes of Health, Bethesda, MD, USA).

All microscopy images were taken with 40x objective magnification (CFI Super Plan Fluor ELWD, 0.6 in N.A.).

### 2.6. ATP Measurements

ATP content was quantified by bioluminescence assay measuring light output from luciferin-luciferase reactions [26]. To evaluate extracellular ATP content, the cell density was adjusted to about 3.5 × 10^4^/mL. 100 *μ*L aliquots of dispersed cell suspensions were placed in 1.5 mL Eppendorf tubes for HMC-1 cells or into 96-well plate for isolated cells. After equilibration for 2 h in the incubator, all samples were divided into nonirradiated control and irradiated groups exposed to laser light for different irradiation time periods. To measure ATP level in the samples, 100 *μ*L luciferin-luciferase assay mix that was diluted with dilution buffer and was adjusted to isotonicity with mannitol was added to each sample, and light emission was measured immediately by luminometer (GloMax 20/20, Promega, Madison, Wisconsin, USA, for HMC-1 cells or Synergy Mx, BioTek, Winooski, USA for isolated cells).

To determinate the intracellular ATP content, HMC-1 cell density was adjusted to about 7 × 10^4^/mL. 50 *μ*L aliquots of dispersed cell suspensions were transferred into 1.5 mL Eppendorf tubes. 50 *μ*L phenol-red-free IMDM and 100 *μ*L somatic cell ATP-releasing reagents were added to each aliquoted 50 *μ*L sample. After briskly swirling, 100 *μ*L mixtures were transferred to each 1.5 mL Eppendorf tube containing 100 *μ*L luciferin-luciferase assay and light emission was measured immediately by luminometer, as above. Manipulation was gentle to avoid mechanical cell stimulation. Luciferin-luciferase luminescence was calibrated versus ATP standards before and after all sample measurements. Intracellular and extracellular ATP is reported in fmoles/cell.

### 2.7. Data Analysis

The data were analyzed by ORIGIN software (OriginLab, Northampton, MA, USA) and expressed as averages ± SEM. The *n* values give the number of measurements obtained from different samples of cells; the *N* values present the number of independent experiments. Differences between sample averages were compared by one-way ANOVA, and *P* < 0.05 was considered to represent statistically significant difference.

## 3. Results

### 3.1. Laser Irradiation Enhances the Extracellular ATP Content of HMC-1 Cells

**Figure 1:**
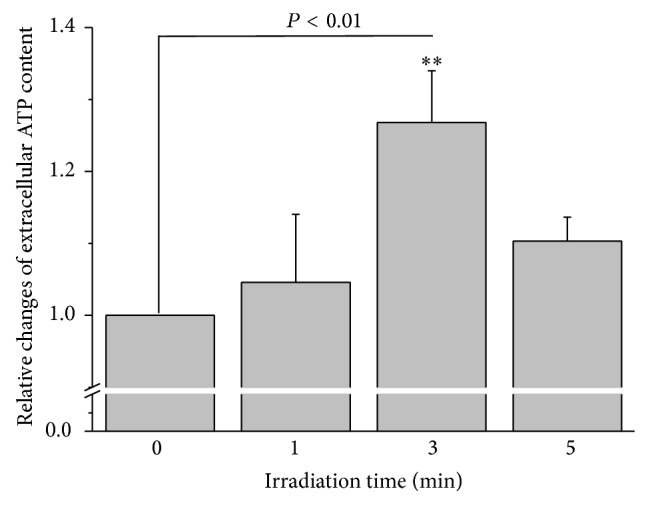
Extracellular ATP content of HMC-1 cells in response to 657 nm and 0.28 W/cm^2^ laser irradiation. Relative changes of extracellular ATP level of HMC-1 cells induced by laser irradiation averaged for respective irradiation times of all samples. The data represent averages ± SEM from *N* = 3-4 independent experiments. ^**^ *P* < 0.01, compared to the preirradiation value.

In control HMC-1 cell suspension samples of 3.5 × 10^4^ cells/mL the extracellular ATP content was 0.41 ± 0.06 fmoles/cell (*n* = 27). ATP content increased progressively with irradiation times of 1 to 3 min. Relative extracellular ATP increase induced by 3 min irradiation was 26.8 ± 7.2% of control (*P* < 0.01, *n* = 8; Figure 1), and it was blocked by 100 *μ*M NEM, an inhibitor of exocytosis (see Table 2). This indicates that the elevated extracellular ATP originated from a cell regulated release process and not from cell death.

It is interesting that the response tended to decline with longer irradiation time of 5 min (with an increase of only 10.3 ± 3.3% compared to control, *n* = 9). This phenomenon has been described as the biphasic dose response to low-level light irradiation discovered in 1985 [27]. It was confirmed in numerous subsequent reports that biostimulation can be obtained at weaker doses, while stronger doses result in bioinhibition. This phenomenon is frequently referred to as Arndt-Schultz law [28].

### 3.2. Laser Irradiation Promotes ATP Synthesis in HMC-1 Cells

**Figure 2:**
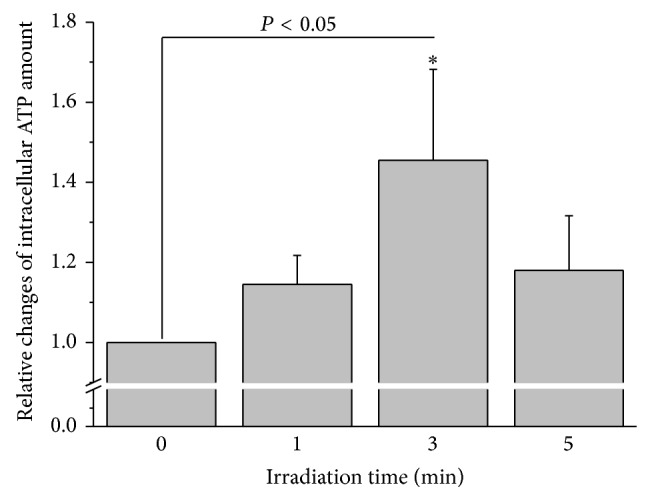
Changes of intracellular ATP content in HMC-1 cells in response to 657 nm and 0.28 W/cm^2^ laser irradiation. Relative changes of intracellular ATP content in HMC-1 cells induced by laser irradiation averaged for the respective irradiation times of all samples. The data represent averages ± SEM from *N* = 3–7 independent experiments. ^*^ *P* < 0.05, compared to the preirradiation value.

Extracellular ATP content depends on three metabolic steps: synthesis, release, and hydrolysis. In various cultured cell types, red and NIR-light irradiations have been reported to promote ATP synthesis [29, 30] that is indexed by intracellular ATP content. Thus in our study, somatic ATP content was assessed. Intracellular ATP in nonirradiated control HMC-1 cells amounted to 14.2 ± 1.8 fmoles/cell (*n* = 30). It increased slightly during the first minute of irradiation and was significantly enhanced, by 45.5 ± 22.6% compared to control values, after 3 min exposure (*P* < 0.05, *n* = 15; Figure 2). Similar to the extracellular ATP responses, 5 min irradiation induced a smaller increase of somatic ATP content (by 18.0 ± 13.7% compared to control, *n* = 9). The coherent response of intracellular with extracellular ATP content may suggest that ATP synthesis partially contributes to elevated external ATP content during irradiation.

### 3.3. Laser Irradiation Increases [Ca^2+^]_i_ in HMC-1 Cells

**Figure 3:**
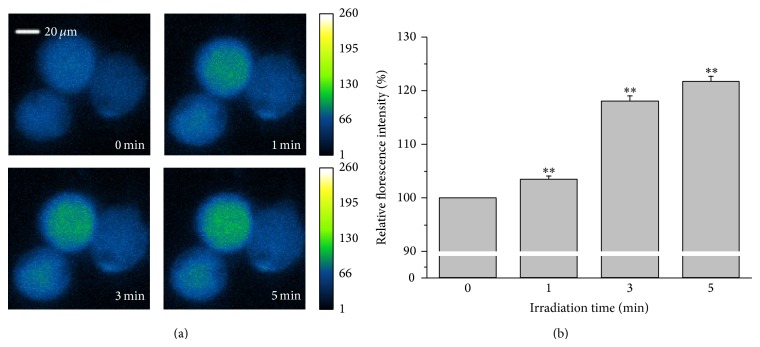
Response of [Ca^2+^]_i_ in HMC-1 cells induced by 657 nm and 0.28 W/cm^2^ laser irradiation. (a) Representative MCs before and after irradiation. Irradiation times are given in the lower right corners. The pseudocolor calibration bar on the right illustrates [Ca^2+^]_i_ (in relative units). (b) Quantitative analysis from several sets of cells normalized to basal [Ca^2+^]_i_-dependent fluorescence before irradiation (=100%). Prolongation of laser irradiation enhanced fluorescence intensity in HMC-1 cells. The data represent averages ± SEM from *n* = 15–17 cells. ^**^ *P* < 0.01, compared to control.

Usually, [Ca^2+^]_i_ elevation is a trigger signal for exocytotic ATP release [31]. Therefore, the response of [Ca^2+^]_i_ to irradiation was assessed. We found that laser irradiation significantly elevated [Ca^2+^]_i_ in HMC-1 cells (*n* = 15–17 cells, *P* < 0.01). An example experiment is shown in Figure 3(a), which illustrates the increase in Ca^2+^ fluorescence at different irradiation times. Increase of relative fluorescence intensity became apparent after 1 min irradiation and amounted to 3.5 ± 0.6% (*n* = 17 cells, *P* < 0.01) of the controls. It rose to 18.0 ± 1.0% (*n* = 15 cells, *P* < 0.01) and 21.5 ± 1.1% (*n* = 15 cells, *P* < 0.01) with irradiation times of 3 and 5 min, respectively (Figure 3(b)). In order to judge whether irradiation-induced [Ca^2+^]_i_ elevation correlates with the ATP release, intracellular free Ca^2+^ in HMC-1 cells was chelated by BAPTA. The results showed that pretreatment of HMC-1 cells with 25 *μ*M BAPTA blocked the extracellular ATP rise induced by 3 min irradiation (Table 2). This suggests that the extracellular ATP level depends on [Ca^2+^]_i_-dependent ATP release.

### 3.4. Laser Irradiation Activates Wild-Type MCs

**Figure 4:**
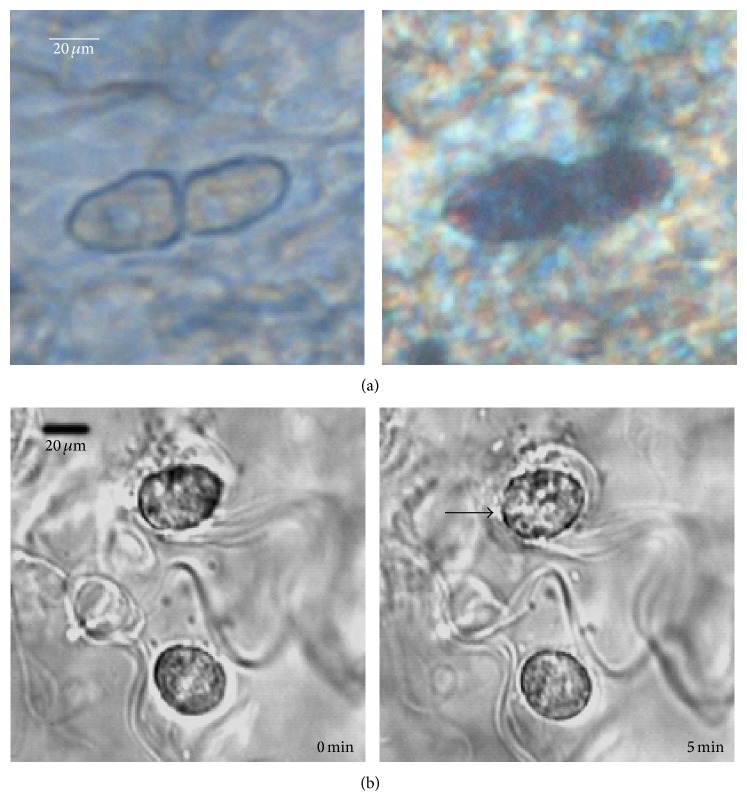
Response of MCs in rat tissue slices to 657 nm and 0.28 W/cm^2^ laser irradiation. (a) MCs in connective tissue slices isolated from rat skin. The left graph shows their smooth and complete plasma membrane. The right graph presents the same MCs stained by 0.5% TB. (b) MCs in tissue slices showed smooth cell membranes before irradiation (left graph). Some MCs started to degranulate once they were exposed to irradiation. The cell membrane became rough, and some black granules were seen scattered around the cell (right graph). The arrow points to degranulating cell. Irradiation times are given in the lower right corners.

**Figure 5:**
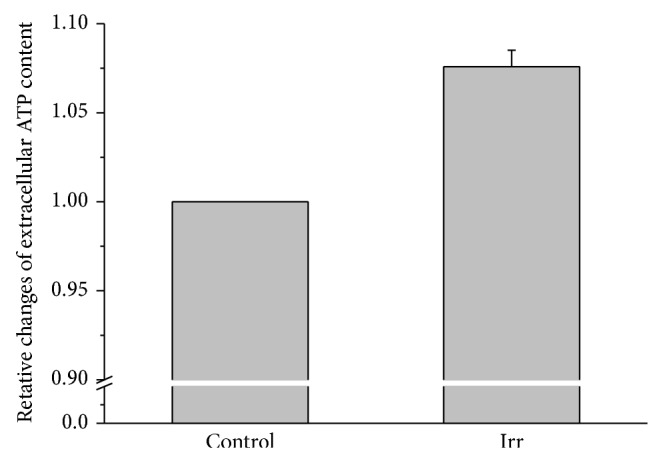
Extracellular ATP content of rat peritoneal MCs in response to 657 nm, 18 mW/cm^2^, and 21 J/cm^2^ laser irradiation. Changes of extracellular ATP content of rat peritoneal MCs induced by laser irradiation averaged for respective irradiation times of all samples. The data represent averages ± SEM from *n* = 12-13.

In order to confirm the effects of red-laser irradiation on wild-type MCs, we measured the response of skin MCs to irradiation* in situ*. Connective tissue slices isolated from the lateral side of the rat tibia contained MCs at high density [32].  Figure 4(a) shows two MCs in a slice section; resting cells exhibited smooth and complete membranes. Identification of MCs was confirmed by staining with 0.5% TB. Once the slices were exposed to red-laser irradiation for 5 min, some MCs in these slices started to degranulate, displaying rough membrane and releasing granules that scattered around the cells (Figure 4(b)). Since peritoneal MCs are of the connective tissue phenotype as skin MCs and the available number of isolated peritoneal MCs is much higher than skin MCs, rat peritoneal MCs were taken to determinate the effects of red-laser irradiation on ATP mobilization. Similar to the responses observed with HMC-1 cells, 21 J/cm^2^ irradiation of rat peritoneal MCs also showed tendency of enhanced extracellular ATP content, although the increase did not reach statistical significance (Figure 5).

### 3.5. Laser Irradiation Attenuates Extracellular ATP Content of Rat DRG Neurons by Modulation of Ecto-ATPase Activity

**Figure 6:**
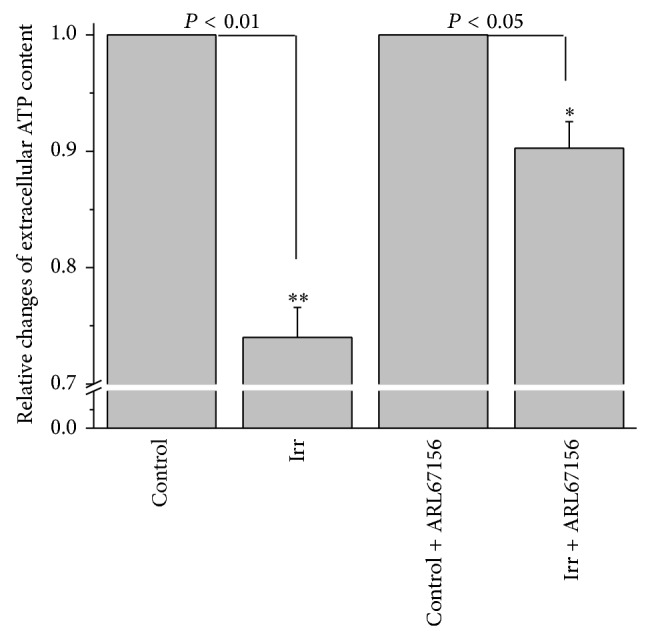
Extracellular ATP content of rat DRG neurons in response to 657 nm, 18 mW/cm^2^, and 21 J/cm^2^ laser irradiation. Relative changes of extracellular ATP level of rat DRG neurons induced by laser irradiation averaged for respective irradiation times of all samples. The data represent averages ± SEM from *N* = 3–7 independent experiments. ^**^ *P* < 0.01 compared to the preirradiation value; ^*^ *P* < 0.05 compared to the preirradiation in the presence of ARL 67156 value.

For nonirradiated neurons, extracellular ATP content was 0.8 ± 0.1 fmoles/cell (*n* = 28). In contrast to MCs, extracellular ATP level was attenuated by 26.0 ± 2.6% in DRG neurons at 21 J/cm^2^ irradiation (*n* = 27, *P* < 0.01, Figure 6). To eliminate the possibility that ATP outside neuronal cells could be degraded by the laser irradiation, the effect of light on ATP was evaluated. The results showed that ATP was not affected by laser irradiation (Table 2). Considering that multiple ecto-ATPases are expressed on DRG neurons [33], we wondered whether the suppressive effect resulted from modulation of ecto-ATPase activity by irradiation. Figure 6 illustrates the response of extracellular ATP content of DRG neurons to irradiation in the presence of the nonspecific ecto-ATPases inhibitor ARL 67156. 100 *μ*M ARL 67156 partially attenuated the irradiation-induced inhibition of extracellular ATP (*n* = 15, *P* < 0.05, Figure 6). This suggests that at least to some extent the irradiation reduced external ATP level of DRG neurons by stimulating the ecto-ATPases activity. The mechanisms of the remaining suppressive effects in the presence of ARL 67156 need further exploration.

## 4. Discussion

*In vivo* MCs have been demonstrated to play a role in LLLT for anti-inflammatory [3], wound healing [7], as well as pain relief [8]. Not only the total number of MCs but also degranulation ratios were increased by red-laser irradiation [6]. In* in vivo* tests, increasing degranulation [12, 13], histamine release [34, 35], and [Ca^2+^]_i_ [12–14, 34, 35] as well as whole-cell membrane currents [12] were demonstrated in MCs irradiated by green-, blue-, or red-laser. Extracelluar ATP is an important autocrine/paracrine mediator to modulate system functions in various tissues by binding to P2 receptors. Red-laser irradiation-induced elevation of extracellular ATP level of MCs has been illustrated in our previous work [14]. In the present study, we confirmed such potentiated effects and demonstrated dose dependency. Extracellular ATP content is influenced by several processes, including synthesis, release, and hydrolysis. In our study, the irradiation-induced coherent response of intracellular and extracellular ATP content suggests that the stimulating effects of irradiation on extracellular ATP can partially be attributed to enhance ATP synthesis. Promoting ATP synthesis by red and NIR-light irradiation has been reported in various cultured cell types [29, 30]. The ATP synthesis depends on the state of the cellular respiratory chain. The absorption of monochromatic light by components of the cellular respiratory chain, such as cytochrome c oxidase, has been reported [35, 36]. Besides providing more ATP to be loaded into the secretory vesicles, increased ATP synthesis also will facilitate ATP-driven transporters such as Ca^2+^ and Na^+^/K^+^ ATPase. Whether the respiratory chain is involved in the processes described in our work needs further investigation. The idea of “photo-switched” ligands that modulate the membrane channels gating has been put forward [37, 38]. This might be another mechanism underling in our researches.

Our results also revealed that irradiation could elevate [Ca^2+^]_i_ in MCs, which is required as the trigger signal for ATP release [31], leading to increased extracellular ATP level. Our experiments with BAPTA-loaded cells provided further evidence that the extracellular ATP level depends on the [Ca^2+^]_i_ rise. Since extracellular ATP is the endogenous agonist of P2X receptors which allow extracellular Ca^2+^ to enter the cells [19], and MCs themselves express P2X receptors [39], we hypothesize that the ATP secreted from MCs could produce positive feedback by autocrine activation of the P2 receptors. Such a mechanism could amplify irradiation effects in local areas to treat local disorders, for example, to act in anti-inflammatory reactions [3] in wound-healing [7], or in local pain relief [8].

Like the subcutaneous MCs, peripheral neuritis in skin is also in a depth that can be reached by irradiation during LLLT [40]. In our work, irradiation attenuated extracellular ATP content of DRG neurons, which is opposite to the response of MCs. The depressive effects of irradiation on peripheral nerve system have been reviewed [41]. Since soluble and membrane-attached ecto-ATPases, that hydrolyze extracellular ATP, exist in peripheral nervous system, including DRG peripheral endings [33], modulation of ecto-ATPases of DRG neurons by irradiation could explain the effect on extracellular ATP levels. In our work, 100 *μ*M ARL 67156 abolished the irradiation-induced depression extracellular ATP of DRG neurons suggesting an upregulation of ecto-ATPases on DRG neurons. The ecto-ATPases hydrolyze extracellular ATP into AMP, the precursor of adenosine which is a vital mediator involved in pain relief by binding to A1 receptors [42, 43]. The peripheral nervous system is the main domain of purinergic receptors, including A1 receptors [19]. Thus modulation of ecto-ATPases by irradiation might be one of the mechanisms underlying analgesic effects of LLLT.

MCs interact with nerve endings morphologically [17] and functionally [18]. There exists crosstalk via purinergic signals between them [20]. Concerning the opposite responses to irradiation in MCs and neurons, we hypothesize that stimulated ecto-ATPase on DRG neurons could hydrolyze part of the extracellular ATP released by the MCs during LLLT.

## 5. Conclusion and Summary

Low-level red-laser irradiation of MCs and DRG neurons has opposite effects on extracellular ATP content. Irradiation of MCs enhanced extracellular ATP level in a dose-dependent manner, which is brought about by increased intracellular ATP synthesis and [Ca^2+^]_i_. For DRG neurons, irradiation depressed the extracellular ATP content due to upregulation of ecto-ATPases. The opposite responses of these two cell types indicate complex mechanisms underlying LLLT that need further investigations.

## Figures and Tables

**Table 1:** Laser system and parameters.

Laser	Parameter
Device	Semiconductor (Model: SB2007047, Shanghai University of TCM, Shanghai, China)
Wavelength (nm)	657
Power output (mW)	35
Mode	Continuous
Irradiated area (cm^2^)	0.126
Power density (W/cm^2^)	0.28
Exposure time (min)	1–5
Dose (J/cm^2^)	17–85

**Table 2:** The response of extracellular ATP contents to laser irradiation in presence of selected reagents.

Reagents (concentration)	Action	[ATP]_o_ in Irr/[ATP]_o_ in control	Conclusion
NEM (100 *μ*M)	Exocytosis blocker	0.96 ± 0.08 (*n* = 8)	No [ATP]_o_ increased once exocytosis was blocked
BAPTA-AM (25 *μ*M)	[Ca^2+^]_i_ chelating agent	1.01 ± 0.13 (*n* = 5)	No [ATP]_o_ increased once [Ca^2+^]_i_ was chelated
ARL67156 (100 *μ*M)	Nonspecific ecto-ATPases inhibitor	0.9 ± 0.02 (*n* = 15)^*^	Inhibition of [ATP]_o_ was greatly attenuated when the ecto-ATPases were inhibited
ATP standards (10 pmoles)	—	1.02 ± 0.06 (*n* = 4)	Extracellular ATP was not affected by laser irradiation

Data are averages ± SEM. Values are relative to control.

[ATP]_o_ presents extracellular ATP levels.

^*^ *P* < 0.05, compared to the control.

## Abbreviations

ARL67156: 6-N,N-Diethyl-*β*-*γ*-dibromomethylene-D-adenosine-5′-triphosphate trisodium salt hydrate FPL 67156
BS: Bath solution
BAPTA-AM: 1,2-Bis(2-aminophenoxy) ethane-N,N,N′,N′-tetraacetic acid tetrakis (acetoxymethyl ester)
[Ca^2+^]_i_: Intracellular calcium activity
DRG: Dorsal root ganglia
HBSS: Hank's balanced salt solution
HMC-1: Human mast cell line-1
LLLT: Low-level-laser therapy
NEM: N-Ethylmaleimide
MCs: Mast cells
NIR: Near-infrared
TB: Toluidine blue.
